# Iron nanoparticles – biodistribution in the chicken embryo model

**DOI:** 10.1016/j.bbrep.2026.102567

**Published:** 2026-03-30

**Authors:** Désirée Schibler, Anna Landsmann, Petra Wolint, Oscar Cipolato, Fabian Starsich, Pietro Giovanoli, Inge Herrmann, Andreas Boss, Johanna Buschmann

**Affiliations:** a Department of Plastic Surgery and Hand Surgery, University Hospital Zurich, Zurich, Switzerland; b Kantonsspital Baden, Im Ergel 1, Baden, 5404, Switzerland; c Laboratory for Nanomaterials in Health, Department of Materials Meet Life, Swiss Federal Laboratories for Materials Science and Technology (Empa), Lerchenfeldstrasse 5, 9014, St.Gallen, Switzerland; d Ingenuity Lab, Balgrist University Hospital, Forchstrasse 340, Zurich, 8008, Switzerland; e Nanoparticle Systems Engineering Laboratory, Department of Mechanical and Process Engineering, ETH Zurich, Sonneggstrasse 3, Zurich, 8092, Switzerland; f Faculty of Medicine, University of Zurich, Rämistrasse 71, Zurich, 8006, Switzerland; g GZO AG Spital Wetzikon, Wetzikon, Switzerland

**Keywords:** Chicken embryo, Biodistribution, Nanoparticles, Magnetic resonance imaging, Histology, Energy-dispersive X-ray spectroscopy

## Abstract

**Objective:**

Magnetic nanoparticles (MNPs) show promise in many biomedical applications, including magnetic resonance imaging (MRI). Iron carbide compounds are good theragnostic contrast agents. Being nanosized might enable passage through a placenta, posing risks to embryonic development. This study investigates the biodistribution of intravenously injected carbon coated iron nanoparticles (ICNP) in chicken embryos.

**Materials and methods:**

Carbon coated iron nanoparticles (300 μg or 600 μg) were injected into a blood vessel on the chorioallantoic membrane (CAM) of chicken embryos (stages 23 to 26) according to Hamburger-Hamilton (H&H). 24 h after ICNP injection, embryos were formalin-fixed and imaged with T2-weighted MRI. Eighteen embryos (three per H&H stage 24–26 and dosage) were further analysed for ICNP deposits via MRI, histology, scanning electron microscopy (SEM) and X-ray spectroscopy (EDX).

**Results:**

Strong signal void artefacts were observed when treated with 600 μg and more pronounced with advancing stages. Deposits were prominent around the vascular system, however all developing organs showed ICNP deposits. Histological and SEM microscopy confirmed inter- and intracellular ICNP uptake in the developing brain. Iron was quantitatively verified in spots of histological sections by EDX. The overall tissue architecture remained intact.

**Conclusion:**

The ICNPs are distributed into most embryo organs and cells without causing damage to the tissue and cells. Their potential to affect embryogenesis warrants further long-term investigation. If proven safe, ICNPs may be used as effective theragnostic agents in MRI as well as for personalized medicine because they can be functionalized with specific proteins or antibodies tailored at patient-specific targets.

## Highlights

• Iron carbide nanoparticles (ICNP) functionalized with streptavidin were successfully injected into the living chicken embryo capillaries to study biodistribution at Hamburger-Hamilton (H&H) stages 24 to 26.
• ICNP distribution was assessed by MRI, histology and EDX. Biodistribution of ICNP imaged with MRI was confirmed with histology.
• ICNP spots in histological sections exhibited increased iron, as assessed by EDX, confirming the iron content of the ICNPs.

## Graphical abstract

The biodistribution of theragnostic carbon coated iron nanoparticles with linked streptavidin was assessed in the chicken embryo model at Hamburger Hamilton stages 24 to 26. Using MRI, nanoparticle deposits were found in the abdominal organs and the brain. This was confirmed by histology and EDX analysis. Such nanoparticles may be used as effective theragnostic agents in MRI as well as for personalized medicine because they can be functionalized with specific proteins or antibodies tailored at patient-specific targets.

**Figure:**
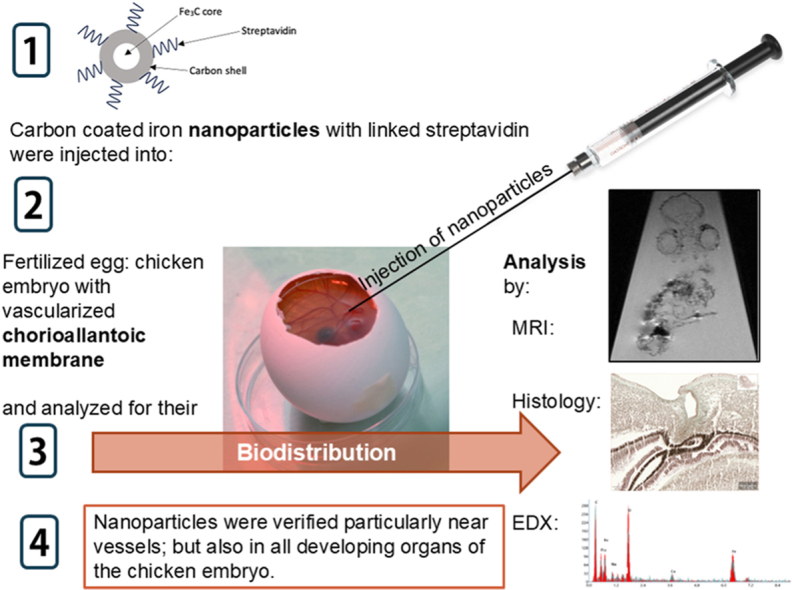

**Table:** Abbreviations

BBB	Blood-brain barrier
CAM	Chorioallantoic membrane
CBS	Circular backscatter detector
CNS	Central nervous system
EMPA	Swiss Federal Laboratories for Materials Science and Technology
ETD	Everhardt-Thornley detector
Fe_3_C(@C)	(Carbon coated) Fe_3_C Iron-Carbide
FoV	field of view
H&H	Hamburger-Hamilton
HR-FSE	high-resolution fast spin echo sequence
ICNP	Iron carbide nanoparticle
IONP	Iron oxide nanoparticle
MNP	Magnetic nanoparticle
MRI	Magnetic resonance imaging
NP	Nanoparticle
PG	Pegylated
RES	Reticuloendothelial system
TE	Echo time
TR	Repetition time
UTE	Ultra short echo time
SEM	Scanning electron microscopy

## 1 Introduction

Magnetic nanoparticles (MNPs) have the potential to serve in a wide array of biomedical applications, especially in all magnetic related fields such as Magnetic Resonance Imaging (MRI) [1], blood purification by magnetic separation [2], magnetic hyperthermia cancer treatment [3] and magnetofection [4] among others. The magnetic component used in MNPs is in this case often iron, due to its higher biocompatibility than for example nickel and cobalt [5,6]. As another advantage besides being more biocompatible, iron can serve as a Fenton reaction agent for theragnostic purposes by generating hydroxyl radicals, when encountering hydrogen peroxide often found in the microenvironment of tumours [7].

Iron oxide nanoparticles (IONPs) have been favoured in biomedical applications because of their modifiable magnetic properties and biodegradability and are considered nontoxic [8]. Another formulation has however gained increasing attention over the years: Iron carbide nanoparticles (ICNPs).

The ICNPs profit from the chemical properties of iron and the favorable characteristics of carbon, such as its mechanical strength and inertness [8]. Comparing iron carbide to other combinations, the intermetallic compound with carbide persuades with a higher magnetic susceptibility than iron oxide, making it potentially a better contrast agent for MRI [3]. Other advantages of iron carbide include a high electrical conductivity, leading to probable higher heating capacity in hyperthermia treatments [9]. A unique benefit from adding carbon to the blend, is its ability to transform the absorbed energy from near-infrared light into ultrasound or heat. This so-called near-infrared light responsive performance is used for photoacoustic tomography and photothermic therapy [10]. The combination of iron and carbide heightens the corrosion resistance, leading to a longer lifetime and decreased toxicity in physiological surroundings, resulting in an overall higher biocompatibility [7].

The nanoparticles (NPs) used in this study are carbon coated iron nanoparticles (ICNPs) with a with a pegylated streptavidin binding site (ICNPs) [2]. The carbon coating further improves the stability and resistance against corrosive agents, allover adding to the biosafety [11]. Apart from protection, the coating also allows for tailored functionalization depending on the intended purpose [12].

While much research time has been devoted to the development and optimization of the magnetic properties of MNPs [13], detailed biodistribution studies are still lacking to further address efficacy and safety for clinical use [8]. Complications, such as unintended accumulation in other than the target tissues need to be examined [14], before they can effectively be used in patients [15].

One way to shed light on the biodistribution of iron based MNPs is the use of MRI, as this imaging tool is already routinely used to visualize pathological iron deposits appearing in diseases such as hemochromatosis [16].

MNPs with an ability to reduce T_2_ relaxation times can be used as a negative contrast agent in MRI when T_2_-weighted [8]. IONPs have been used quite commonly for that purpose, however with the downside of a low transverse relaxivity (r_2_), in turn leading to a lower sensitivity for diagnosis [8]. Some success at better visualization has been seen using ultra short echo-time T2∗ mapping to detect injected IONPs in mice using MRI [17]. In general, ideal MNPs possess high r_2_ values, which are largely dependent on the magnetization M_s_ of a material, as can be deduced by the quantum mechanical outer sphere theory [8]. The ICNPs used in this study, Fe_3_C with octahedral interstices, have a larger M_s_ value than IONPs, namely 140 emu g^−1^ compared to 92 emu g^−1^ attributed to IONPs [12]. As a consequence, even more pronounced hypo-intensities on T_2_ weighted MRI can be expected when compared to IONP based contrast agents [18].

When it comes to testing drug safety, the developing embryo has been one of the ignored patient groups [19], even though embryos are more vulnerable to xenobiotics than adults [20]. Being nanosized, MNPs are able to pass through the placental barrier, potentially leading to developmental issues or even death in the embryo. Various studies discuss the adverse effects of NP exposure during pregnancy, bringing about all kinds of toxicities in the developing organism [20].

Due to ethical concerns, studies on human embryos are impossible. To conduct studies in accordance with the 3Rs principle, namely Replacement, Reduction and Refinement, chicken embryos have however gained an increasing interest over the years [21].

Chicken embryos may allow to study the effects during pregnancy and potentially also in adult humans.

Since the biodistribution and toxicity of ICNPs has not been widely tested in model organisms, especially no embryological model organisms, we focused our study on the following three research questions.

(i) Do ICNPs circulate in the chicken embryo and deposit in specific tissues?
(ii) Where are the supposedly deposited nanoparticles detectable with an MRI T2 sequence?
(iii) Can we confirm the presence of ICNPs in chicken embryonic tissue by quantitative EDX?

## 2 Material and methods

### 2.1 Nanoparticle production

**Fig. 1:**
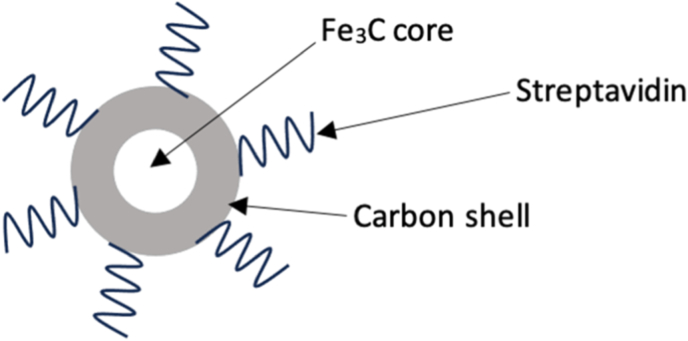
Pegylated carbon-encapsulated ICNPs with a streptavidin bound to the carbon shell.

The carbon coated iron nanoparticles ICNPs (Fig. 1) were purchased from Sigma Aldrich [12]. These MNPs with octahedral interstices and a mean diameter of 40 - 60 nm, a saturation magnetization = 140 emu g^−1^ and a specific surface area of 30 m^2^g^-1^, have been washed six times over the course of 24 h using a concentrated mixture of HCl_(aq)_ (Fluka, puriss) and deionized water (Millipore, resistivity = 18.2 M Ω cm) in a 2: 1 ratio. After the incompletely coated NPs had dissolved, they were again washed with millipore water for three times to remove the acid. In the end, they were dried in a vacuum oven at 60 °C. Streptavidin (ANAWA, Wangen-Zurich, Switzerland) was bound to the ICNP carbon coat according to previously published protocols [2]. It served as a model protein for any other protein that may be linked to the ICNP carbon coat in the future.

### 2.2 Preparation of suspension and injection of nanoparticles

The ICNP suspension with a concentration of 10,000 μg/mL was diluted in Trypan blue at a ratio of 3:2 (3 ICNP: 2 Trypan blue) resulting in a concentration of 6 μg/μL. The Trypan blue was used for better visualization of the distribution in the vascular system during injection. 100 μL of the suspension was injected in the 600 μg embryo cohort, and 50 μL in the 300 μg cohort. To create a homogenous Fe-concentration in the suspension, it was vortexed (JK MS1 Minishaker IKA ®) for 30 s previous to each injection. To minimize the bleeding at the site of injection, a piece of Alginat wadding (Derma Plast ®, IVF Hartmann AG, Neuhausen am Rheinfall, Switzerland) was placed at the puncture site.

### 2.3 Chicken embryo model to study biodistribution

**Fig. 2:**
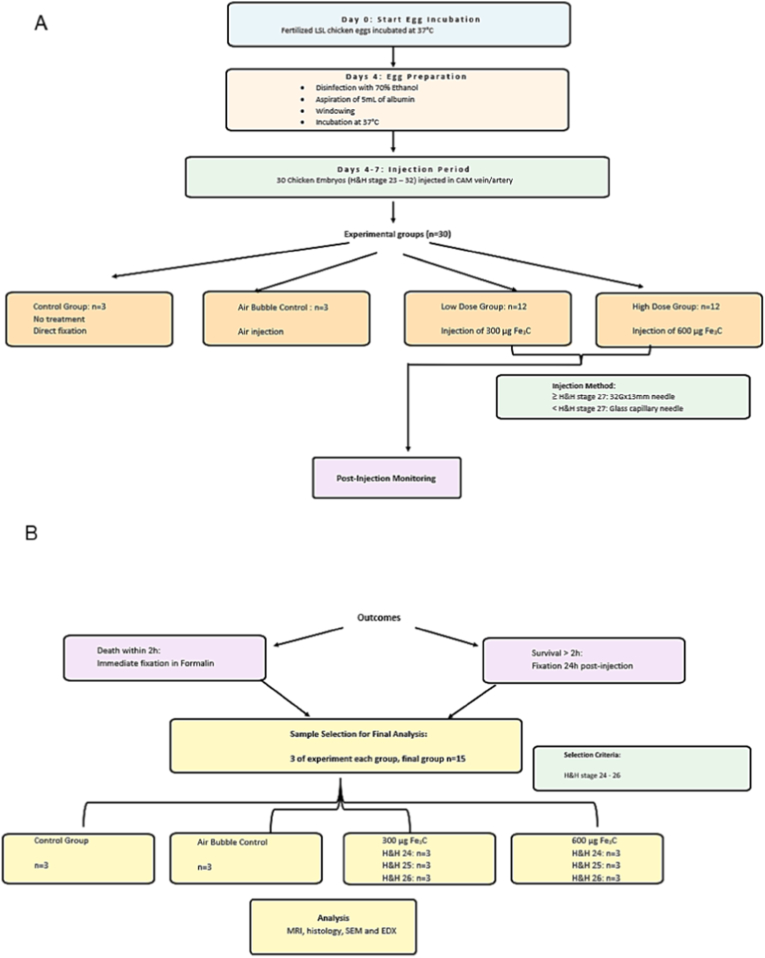
Workflow chart with number of samples per condition; for conditions (**A**) and analyses (**B**).

Fertilized Lowman white LSL chicken eggs were supplied by Animalco AG (Puoltry farming, Schongau, Switzerland). According to the Swiss animal welfare guidelines (TSchV, Art. 112), no IACUC approval is required until the 14th day of development of the chicken embryo. Embryonic day 0 was designated as the day when the eggs were incubated. On day 4, the eggs were disinfected with 70 % ethanol and placed onto a plastic shell. Using a Dremel (300 series), a small slit was cut along the broader end of the egg, through which 5 mL of albumin was aspirated using a syringe. The slit was then sealed with disinfected tape. As a next step, a window was made on top of the eggs, again using the Dremel and tweezers. After placing a plastic lid on the window, the eggs were placed into an incubator (Binder) at 37 °C for another 24 h. Through embryonic days 4 to 7, some of the embryos were used for injection of ICNPs.

irty Chicken embryos (H&H-stage 23-32) **(**SI Fig. 1**)** were injected with 300 μg or 600 μg of ICNPs into an accessible blood vessel on the CAM. A workflow chart is given in Fig. 2.

**Fig. 3:**
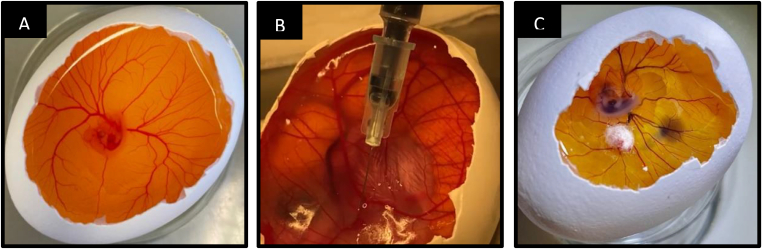
Images of chicken embryos being injected. A: Opened egg with not yet injected embryo, H&H stage 25. B: Opened egg with chicken embryo during injection, H&H Stage 32. C: Opened egg with chicken embryo shortly after injection, the injected suspension being visible as a blue tint inside the cardiovascular system, H&H stage 25. A piece of white Alginat wadding (Derma Plast ®, IVF Hartmann AG) can be seen at the site of injection to stop the bleeding.

At an embryological H&H stage of 27 and upwards, a 32Gx13mm needle (GMS) was used (Fig. 3).

Below that stage, with the blood vessels being too thin and fragile for a 32G needle, a glass needle self-pulled from a glass capillary, was used. Specifically, a glass capillary was elongated by pulling while it was in the flame of a Bunsen burner. After cooling it down to room temperature, it was broken in the middle where it was thinnest, and one part of it was connected to a 32G needle by inserting the needle into the end of the pulled glass capillary. The ICNP solution with a concentration of 10′000 μg/mL was diluted in Trypan blue at a ratio of 3:2 (3 Fe_3_C: 2 Trypan blue) resulting in a concentration of 6 μg/μL. The Trypan blue was used for better visualization of the distribution in the vascular system during microinjection. 100 μL of the solution was injected in the 600 μg embryo cohort, and 50 μL in the 300 μg cohort. To create a homogenous Fe-concentration inside the solution, it was vortexed (JK MS1 Minishaker IKA ®) for 30 s previous to each injection.

After injection, the embryos were incubated for another 24 h, unless they died right after injection. When death occurred within the following 2 h, they were immediately fixated in 4 % formalin. When they survived, they were fixated 24 h after injection. Of the 30 chicken embryos used, for both 300 μg and 600 μg a subset of three embryos each from age 24 to 26 was used for further analysis in this study. Three chicken embryos were not treated at all and fixated directly as a control, another three were injected with air bubbles (Fig. 2). The microinjections were always performed by the same operator, ensuring operator consistency. No blinding or randomization was performed, as the operator both prepared and administered the solutions to the next available chicken embryo without prior allocation to random cohorts. Thus, eggs were randomly attributed to experimental groups.

The fixated embryos were then placed onto a cradle and moved into an animal MRI and later prepared for histology.

### 2.4 Magnetic resonance imaging scan protocol

All examinations were carried out on a Bruker 4.7 T BioSpec 47/40 animal MRI (Bruker BioSpin, Ettlingen, Germany) equipped with a circular polarized 1 H mouse whole body transmitter-receiver radiofrequency coil. First, a gradient-echo localizer was applied in axial, sagittal, and coronal planes to provide an anatomical overview. A T2-weighted high-resolution fast spin echo sequence (HR-FSE) was acquired in transverse, sagittal, and coronal planes using the following image parameters: repetition time (TR) 2646 ms; effective echo time (TE) 45 ms; field of view (FoV) 30 × 30 mm; flip-angle 180°; slice thickness 1 mm; interslice distance 1.5 mm; five averages. The standard reconstruction algorithm of the Bruker HR-FSE sequence was applied for image reconstruction.

### 2.5 Histology, scanning electron microscopy and elemental analysis

The samples where all sliced in the sagittal plane with the cut slicing through the areas where nanoparticle accumulation was seen in the embryo by the naked eye and observed on the MRI: namely the abdominal area and the brain bud including the retina. Using standard histology preparation protocols, the samples were then fixed in 4 % formalin for one day.

Paraffin embedding included dehydration, paraffin-embedding and sectioning into 3 μm thick slices. Before they were stained, paraffin embedded sections were deparaffinized utilizing xylene and rehydrated (descreasing gradient of ethanol).

The 3 μm thick sections were stained with Hematoxylin& Eosin (H&E) and with Masson Goldner Trichrome (MGT). The slides were scanned and analysed using the program QuPath-0.5.1-x64 [22].

Scanning electron microscopy (SEM) was conducted using a TFS Magellan 400 (Thermo Fisher Scientific) scanning electron microscope at ScopeM. The sample was coated with 10 nm of carbon (CCU-010 Carbon Coater Safematic) prior to SEM imaging. Images were acquired at an acceleration voltage of 5 kV, a beam current of 0.10 nA, and a working distance of 4 mm. Secondary and backscattered electrons were detected using an Everhart-Thornley detector (ETD) and a circular backscatter detector (CBS), respectively. Elemental analysis was performed via energy dispersive X-ray spectroscopy (EDX) using an EDAX Octane Super detector. Energy calibration was handled automatically by the EDAX TEAM™ software using the detector's internal calibration routine. For EDX measurements, the microscope was operated at an acceleration voltage of 18 kV, a beam current of 0.10 nA, and a working distance of 10 mm. Both single point and area scans (256 × 192 pixels) were performed to verify the presence of ICNPs. Spectra were processed using the standard model-based background subtraction implemented in EDAX TEAM™ software.

### 2.6 Data analysis

Qualitative data analysis was done by determining the exact location of the ICNP deposits and visually comparing the MRI images and correlating them histologically. Differences in the high dose compared to the low dose were qualitatively assessed. Moreover, EDX analysis was used to confirm ICNP deposits in histological sections.

## 3 Results

### 3.1 Survival

A total of 30 embryos were used in this study. Three were used as a reference without any injection, another three were again used as a reference when injected with air bubbles. Two of the controls injected with air bubbles died immediately after injection, the other one within 24 h. The 24 remaining embryos were injected with relatively high dosages of ICNPs, i.e. either 300 μg or 600 μg of ICNPs, of which 18 embryos were used for further analysis for this study after assessment of the development stages, with 9 for each of the two experimental dosage groups. The high dosages of ICNPs were chosen to simulate a worst-case exposure scenario. Two of these 18 embryos survived 24 h after injection, a total of five died immediately after, two within 2 h and nine sometime within the following 24 h (SI Fig. 2).

### 3.2 Qualitative assessment of scans

**Fig. 4:**
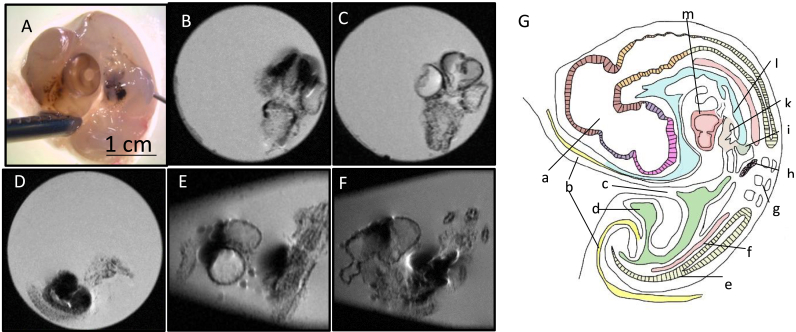
Overview of Embryo 24.A.600 under Microscope and MRI, compared to a schematic ov**erview. A:** Embryo 24.A.600, not yet prepared. Nanoparticle deposits are visible inside the body in brain region, retina, heart and possibly liver. **B:** MRI of head, coronal plane. Contours of eyes and border between diencephalon and mesencephalon are well visible. **C:** MRI of head, coronal plane. Nanoparticles are visible in mesencephalon and presumably along blood vessels. **D**: MRI of embryo mid body tilted axial plane. Deposits are prominently visible in heart and liver right below it. **E:** The two round structures are a cut through the optic cups on both sides. Striped hypointensities correspond to the neural tube. The liver, heart and mesonephros show complete signal loss with artefacts. **F**: MRI of whole body, sagittal plane. Contour of body well visible, signal loss in heart, optic cup and liver. **G**: Schematic overview of the chicken embryo anatomy at day 4 (according to: Virtual Classroom Biology, n.d.) [23]. a: Brain, b: Amnion, c: Mid-gut, d: Allantoic Vesicle, e: Neural tube, f: Dorsal aorta, g: Somite, h: Mesonephros, i: Dorsal pancreas, k: Liver, l: Stomach region and Esophagus, m: Heart.

**Fig. 5:**
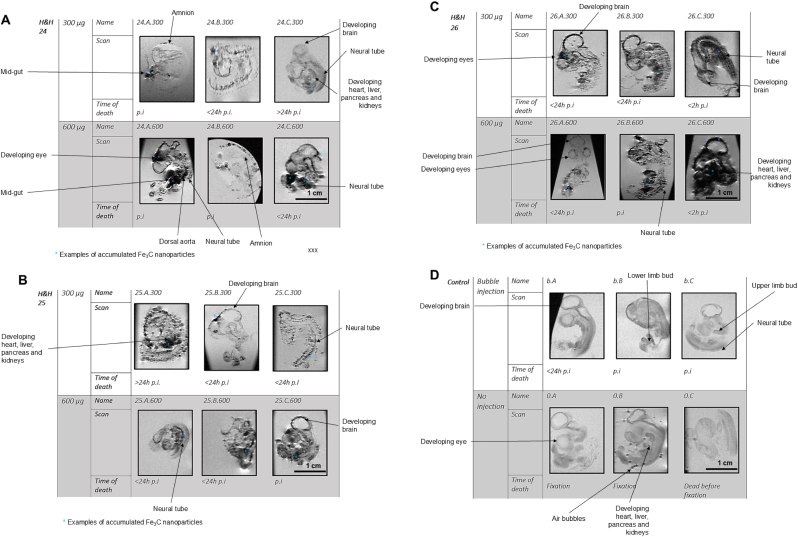
Overview of the MRI scans of 18 embryos in sagittal plane, including the time of death. Signal loss and hypointensities darker than seen in the control group are interpreted as consequences from accumulated ICNPs. Hamburger Hamilton Stage 24 (***A***), 25 (***B***), 26 (***C***) and Control (***D***).

HR-FSE sequences with T2-weighted contrast (TE 45 ms) were acquired, the resulting sagittal images are shown in Fig. 5. An example of an embryo before preparation, as well as a representative of the resulting MRI scans in axial, sagittal and coronal plane are shown in Fig. 4. Comparing the three H&H stages within the same concentration group, a general higher contrast and more pronounced signal loss can be observed the further developed the specimen is, this effect can be seen more so in the lower concentration group of 300 μg. Especially in the chicken embryos treated with 600 μg ICNPs, a complete signal loss to background noise can be observed in most developing organs, a clear differentiation of the abdominal organs is not possible. The brain bud including the optic cup show conspicuous signal loss as well. In the tissues loaded with ICNPs, clear margins cannot be discerned. Scale bar 1 cm presented in image A is the same for all images.

In chicken embryos without nanoparticle injection, the HR-FSE sequences show intermediate intensity of the tissues, with slightly more pronounced CNS structures, as well as the anterior and posterior appendage bud (Fig. 5).

Calculation of contingency coefficients and according p values revealed that there was no significant difference between the MR signal intensities of the abdomen and the CNS, with p = 0.602 (Table 1). For the comparison of the two dosages, there was a significant difference between 300 and 600 μg, with p = 0.021 (Table 2). Further semiquantitative analyses of MR intensities revealed no significant differences, when compared for the H&H stages; for H&H24 versus H&H25 p = 0.767; for H&H24 versus H&H26 p = 0.149; for H&H25 versus H&H26 p = 0.094 (SI Table 1).

**Table 1:** Crosstable comparing semi-quantitatively MRI intensities for two organs, the abdomen and the central nerve system (CNS), respectively.

MR Intensity	Low	Intermediate	High	Very high	Total
Abdomen	3	5	8	2	18
CNS	5	7	5	1	18
Total	8	12	13	3	36

**Table 2:** Crosstable comparing semi-quantitatively MRI intensities for two doses, 300 and 600 μg, respectively.

MR Intensity	Low	Intermediate	High	Very high	Total
300 μg	7	7	4	0	18
600 μg	1	5	9	3	18
Total	8	12	13	3	36

### 3.3 Histology

**Fig. 6:**
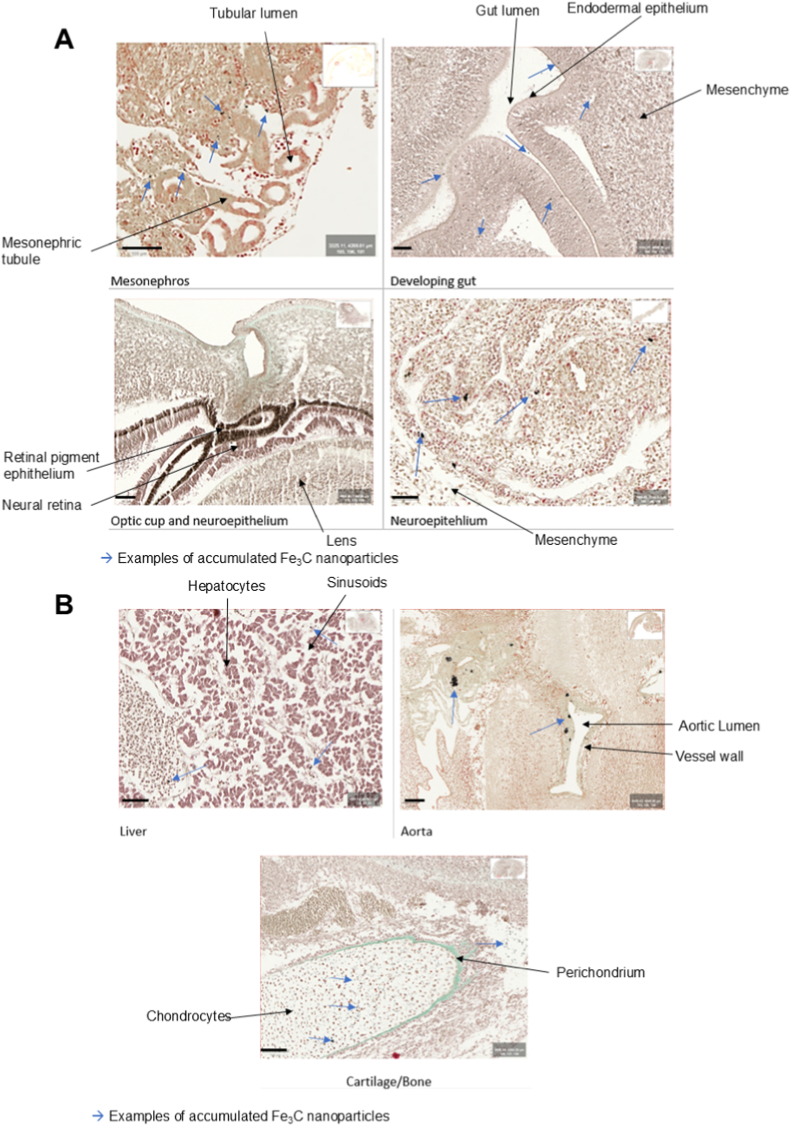
Overview of histologically prepared different tissues, MGT-stained. Examples of ICNP clusters are indicated with a blue arrow. The overall color balance appeared to be of a more grey-brownish quality across all sections. Scale bars: 100 μm (mesonephros; aorta; and cartilage/bone) and 50 μm (optic cup; liver; neuroepithelium and gut).

All samples were stained with Masson–Goldner trichrome (MGT). According to the well established staining characteristics of control MGT preparations without nanoparticle exposure, collagen typically appears green-blue, cytoplasm red-pink, and nuclei dark purple. Our samples however showed a diffuse grayish or brownish hue across various tissues, as well as black aggregates interspersed in most tissues (Fig. 6).

A cross-section of the developing mesonephric tubules shows a greyish discoloration of morphologically intact tubules without signs of necrosis or inflammatoriy infiltrates. Dark, punctate deposits can be found both within and around the tubular epithelium, indicated with a blue arrow. A similar picture of discoloration and interspersed black aggregates can be observed in the neuroepithelium. The neuroepithelium surrounding the optic cup appears even darker, while the developing retina can be discerned as a brown band with a well-preserved overall architecture. No distinct aggregates can be defined there, though they cannot be ruled out either.

Especially in the developing gastrointestinal tract many diffusely scattered grey-black aggregates can be found within the epithelium and in the surrounding tissue. The cell morphology still remained structurally intact and well organized. Similar to the gastrointestinal-tract, small, widely dispersed NP-clusters are found in the developing cartilage of an early appendage bud. Still, no cellular damage or morphological disruption is visible.

Less punctuate clusters could be found in the developing liver, though the tissue exhibited a diffuse brownish discoloration, potentially due to widespread uptake. The liver tissue had a well preserved hepatic architecture with well-defined hepatic cords and sinusoidal spaces.

A section of the aorta next to another prominent vessel show distinct nanoparticle aggregates along the lumen's perimeter an the adjacent mesenchyme. Despite this accumulation, the surrounding tissue maintaines a healthy, organized morphology without significant alterations in cellular density or structure. Across all examined sections, ICNPs can be suspected intracellularly in the form of a diffuse black-brown cellular coloration and accumulation was evident surrounding multiple organ systems, present as discrete interstitial black aggregates.

### 3.4 SEM

**Fig. 7:**
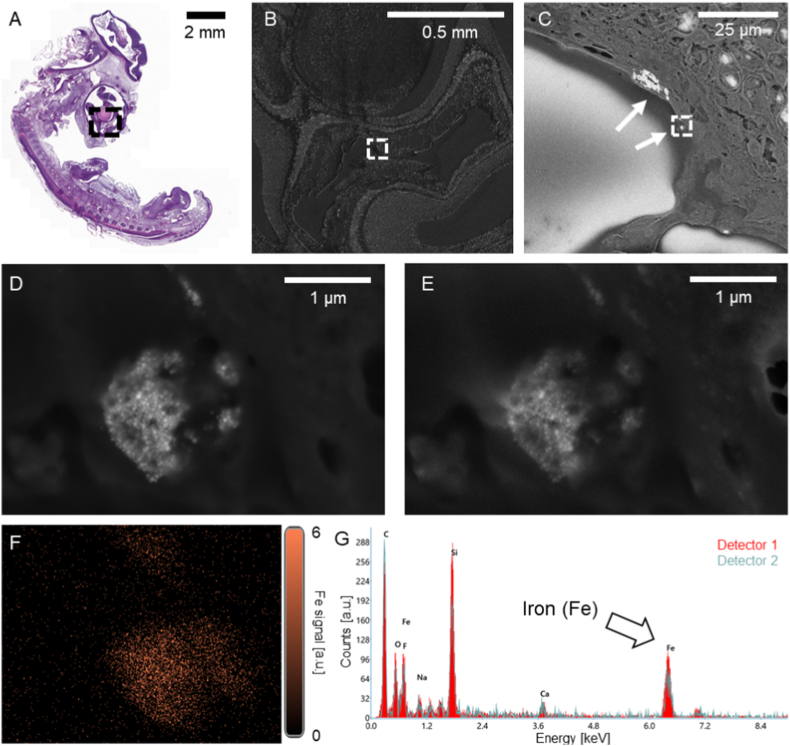
SEM images and EDXS analysis of ICNPs detected in embryonic tissue. H&E stained section of a whole chicken embryo treated with 600 μg of ICNPs (**A**). Dashed black square indicates region chosen for CBS image in (**B**). White dashed square reflects area chosen for magnified view showing clusters of ICNPs, indicated with white arrows (**C**). Further magnification revealing individual nanoparticles within the ICNP cluster, situated in the embryonic tissue (**D**). ETD detector image of the same cluster (**E**). EDX elemental map of iron (Fe) in the region (**F**) and EDX spectrum acquired from the cluster (**G**) with an arrow for the iron peak.

In the overview SEM image taken with an ETD detector, heterogeneously scattered, bright electron-dense aggregates are visible, indicative of nanoparticles (Fig. 7). The brighter imposing band corresponds to the developing brain and retina. Over all, the anatomical structures appear to be preserved.

At a higher magnification, the structural integrity still appears intact, with the nanoparticles mostly clustered close to the cell surfaces but also potentially intracellularly (Fig. 7c, white arrows). The CBS detector confirms the presence of a higher-density material in the location of the bright spots visible through the ETD detector and inside some of the cells.

**Fig. 8:**
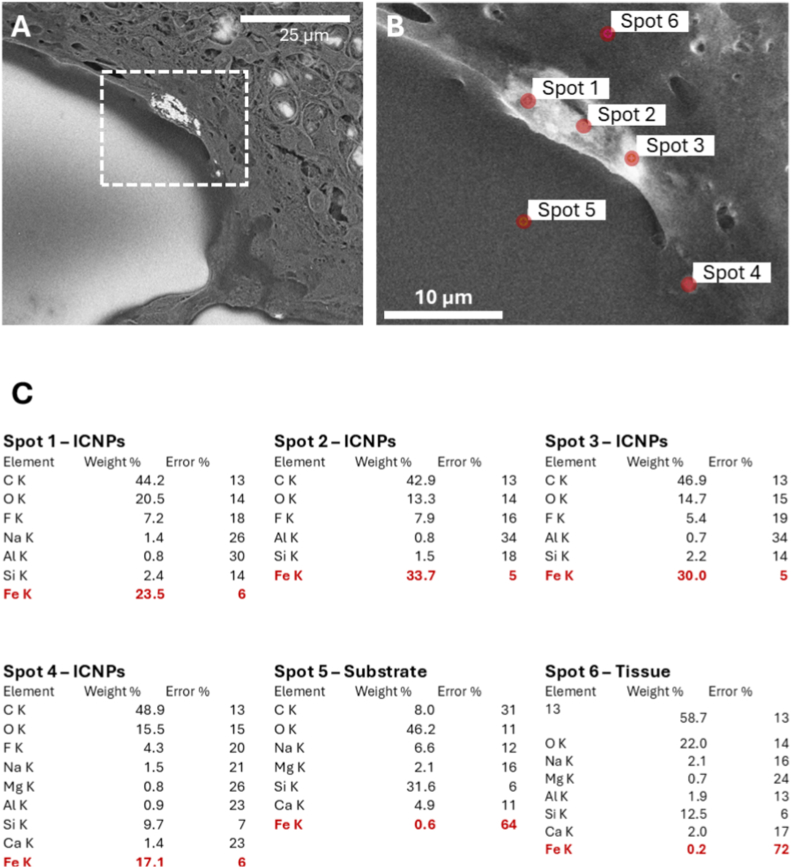
SEM images and EDXS analysis of ICNPs: quantification of Fe at distinct spots. Backscattered image with the investigated area in the white dashed square (**A**). This image is from Fig. 7C. Secondary electron image of the investigated area with the spots where elemental analysis was carried out (**B**). Elemental composition at the various spots showing how the bright areas are indeed ICNPs. The elemental mass percentages are based on the K lines of each element and are obtained processing the spectra using the fitting function provided by the EDAX TEAM™ software (**C**).

Even higher magnification of the SEM images reveal the ICNPs having aggregated. These aggregates show a strong adherence or possible internalization inside the cells. EDXS analysis confirmed the presence of ICNPs by elemental iron (Fe) (Fig. 7f). Elemental composition of distinct spots based on the K lines of each element obtained by processing the spectra with a fitting function provided by EDAX TEAM™ software are shown in Fig. 8. Weight percentage values are for reference (Fig. 8C).

## 4 Discussion

To truly estimate the potential of the in vivo performance of any kind of nanoparticles, a clear understanding of its biodistribution in a living complex organism is crucial. Only after innately targeted tissues and regions of unintended accumulation are identified, predictions of possible applications, clearance mechanisms and side effects can be made [14,24]. The ICNPs used in this study have unique magnetic properties with suggested high biocompatibility and possibility for surface functionalization, making them promising candidates for hyperthermic treatments, magnetic blood purification [2] and drug- or gene delivery and simultaneously potential contrast agents [25].

Human experiments are limited in accordance with ethical guidelines [26]. Particularly when investigating effects on embryological structures, regulations are very strict. Chicken embryos have emerged as a more ethical, easily available, and cost-effective means for testing xenobiotics in a developing organism. Beside effects on embryological tissue, first general impressions on the biodistribution and performance of NPs in a physiological setting can be made and its structure optimized, before being further tested on rodents [27]. A great advantage is also the close resemblance to human embryology in the early developmental stages [28].

In this study, we injected 30 chicken embryos with H&H developmental stages reaching from 22 to 26 with ICNPs. Three of the formalin fixated embryos for each H&H Stage 24, 25 and 26, as well as three controls each with and without bubble injection, were then imaged with a small 4.7 T animal MRI (Fig. 2).

The MRI is an ideal non-invasive imaging tool for a broad visualization of the ICNPs, since this MNP has a high magnetic saturation magnetization (M_s_) of 140 emu g^−1^. As a consequence, hypointensities superior to the more commonly used IONP based contrast agents can be expected on a T2 weighted image, as IONPs only have an M_s_ of 92 emu g^−1^ [12]. The higher the saturation magnetization of a contrast agent, the stronger is the reduction of transverse relaxation [29].

In a study where BALB/c mice were injected with Fe_5_C_2_ ICNPs, which are also of octahedral interstices like the ICNPs used in our study, high signal void artefacts in the liver, even higher than compared to Fe_3_O_4_ IONPs were determined [30]. Our results for the high-dose group indeed show strong void artefacts (Fig. 5) with complete signal loss in the developing abdominal area of most chicken embryos (Table 1). An accumulation inside the liver is suggested in the scans and histologically as well (Fig. 6). A clear demarcation of the liver from other potential developing organs is however not possible on the MRI scans.

A study conducted with IONPs by Boss et al. suggested higher NP concentrations led to stronger reduction of T2∗ [17]. Accordingly, a darker appearance with a more pronounced signal loss hints at an increased T2 relaxation time in the high-dose group as well in this study. The contrast appears higher, with the contours of the body and its developing organs better visible compared to the 300 μg group. A semi-quantiative analysis with a crosstable revealed a significant difference in MR intensities when the low and high dose groups were compared to each other (Table 2). Both groups however gain hypointensities with higher developmental stages, this effect is more apparent in the low dose group, although no significant differences were calculated for the inter-stage comparison of the H&H stages (SI Table 1). Higher H&H stages go along with a more developed vascular system and in turn better distribution of the injected ICNPs. Of course the injection itself was more easily and perhaps more successfully performed in an embryo with better visible vasculature on the CAM.

Some MRI scans show large, bubble-like artefacts, that have a close resemblance to the air-bubbles sometimes seen in the surrounding fluid of our MRI scans. In these images, it is exceedingly difficult to assign the artefact to a specific region. We suspect these artefacts to be due to the larger aggregations of ICNPs found inside or close to a vessel, as well as the intercellular area. After all, in the control group with deliberately injected air-bubbles no such artefacts were observed.

Possible adverse effects of novel materials within a biological system remain a significant barrier to their clinical adoption, especially in vulnerable patient groups such as developing organisms.

One cause of embryo death could be attributed to accidental air injections. In the control group receiving bubble injections, two out of three died immediately post injection. It must be noted that for the further analysed chicken embryo stages H&H 24 to 26, all of our needles used for injection were self-pulled from glass capillaries and then imposed and fixated with a strip of Parafilm® on a 32 G needle without an air-filter, therefore air embolism is a risk. Of the presented embryos, only two out of 18 survived 24h after injection (SI Fig. 2). Potential intravascular air bubbles could unfortunately not be detected with MRI.

During the early trial injection process prior to this study, accumulated ICNP were visible to the naked eye in the vessels spanning across the CAM (SI Fig. 3). It was thus decided to vortex the suspension before injection for 30 s, after which no accumulations could be perceived during injection. However, a formation of smaller clusters not visible without higher magnification cannot be excluded. Histologically, many of the samples showed deposits surrounding blood vessels and ICNP aggregates of various sizes up to 50 μm have been found surrounding blood vessels. Even smaller aggregates ranging from 1 μm to 7 μm might have impacted blood flow. No statements can be made regarding the flow characteristics of our aggregated ICNPs, further studies would be needed to do so.

However, not only the ICNP aggregates themselves could act as a thrombogenic material. A feared risk of injecting a material intravascularly, is the triggering of the blood coagulation cascade, leading to potentially detrimental consequences such as thromboembolism up to disseminated intravascular coagulation. Many carbon-based materials have been shown to influence the coagulation cascade in a prothrombotic way [31]. Bircher et al. have observed slightly reduced clotting times when introducing ICNPs, with the PEGylation of the nanoparticles however decreasing this effect [32]. There were, however, no overt blood clots in our histological samples. It is important to note that the coagulation system in chicken embryos develops later, so this risk of blood clots would not be expected at the H&H stages under view here [33].

Not only clot formation is a risk, however, hemolysis or interaction of the nanomaterial inside the red blood cells leading to decreased oxygen transportation capacity need to be considered and studied further. A study conducted by Herrmann et al. showed no significant influence at a concentration of 1 μg/μL of ICNPs with or without PEG-ylation on either blood clot formation or hemolysis when compared to a control [34]. In contrast, we have injected a suspension at a six times higher dosage (6 μg/μL), which may nevertheless pose risks regarding clot formation or hemolysis. Further studies will be needed to determine the effect of higher concentrations as well as the impact of blood circulation in a vascular in vivo system.

Another confounding effect might be the use of Trypan blue, which facilitated the verification of successful injection. However, several studies indicate a teratogenic and even toxic effect leading to a higher mortality rate [35,36]. So there are indeed many limiting and confounding factors that could have affected the outcome of our pilot study. These factors cannot be ruled out to have had an impact.

Making assumptions about our ICNP nanoparticles based on other similar nanomaterials is difficult, as even small differences in size, coating, functionalization and size of aggregated NPs can have a massive impact on biodistribution and influence on cells.

Apart from the nanoparticle characteristics, the quality of a blood vessel's endothelial barrier plays a pivotal role in its biodistribution. While human vessels in the lung or muscles are characterized by a dense continuous surface, the endothelia in the kidneys are fenestrated and those of the liver and spleen are discontinuous, allowing for a generous passage into the surrounding tissue [15]. Especially in the brain, connections between endothelial cells are very tight, to keep as much unwanted molecules outside as possible [37]. Various efflux mechanisms support the tight junctions of the endothelium [38]. While helpful to keep pathological substances out, it can be a hindrance when trying to target a pathological process inside the brain. Here, the theragnostic potential of NPs comes into play again. Several ways have been investigated to use NPs as carriers across the blood-brain barrier (BBB), for example by functionalizing their surface with a ligand that promotes the uptake [39,40]. A tight BBB is not only important in adulthood, but also essential during embryological brain development. For a long time, it has been assumed that the human brain's barrier was leaky during development and even shortly after birth [41]. Studies have however shown, that as soon as the neural tube closes during development, certain proteins are already unable to enter the brain and efflux transporters appear with further development [38]. In a study focusing on chicken embryos however, it was found that the blood vessels present in neural tissues permitted the passage of Evans blue and horseradish peroxidase into the CNS well up to the 10th day of incubation [42]. Further studies are needed, to determine if this permission of ICNP across the BBB is unique to the species of chicken embryos or can be observed in other embryological organisms as well. In all our MRI scans featuring the brain, retina and neural tube of the chicken embryo, signal void artefacts were visible along the contours of the developing CNS, suggesting an unhindered access of the ICNPs to the brain.

In various samples, ICNPs were histologically found inside the brain tissue, confirming the MRI signal void artefacts seen. When looking at the MGT stained histology samples, what stands out is a noticeable grey or brownish cast in many cells, and punctate black spots where the ICNPs have aggregated. Under the experimental conditions, ICNP exposure did not influence early embryonic tissue architecture histologically. We interpret the grey-brown appearance of our samples as the intracellular uptake of ICNP altering the usual color profile of MGT staining. There is also evidence of intracellular nanoparticle deposition in neural cells in SEM, which has implications for both the therapeutic efficacy and potential cytotoxicity of ICNP. In SEM, no damage or cellular disruptions were observed either, but the discrepancy to the high mortality rate still calls for further investigation.

The possible risks due to accumulated ICNPs inside tissues, especially the neural tissue, depend on how active the NPs are inside the tissue and its cells. An iron overload inside the brain tissue is objectively harmful, as can be seen in diseases like hemochromatosis, thalassemia or long-term blood transfusion therapy which lead to a build-up of iron inside of cells and can also be detected in a T_2_ weighted MRI scan [17].

In a study, Fe_5_C_2_ NPs were treated for 72h in a pH 5.0 buffer solution similar to an acidic lysosome environment and were shown to be partially or fully degraded, with the released iron having the potential to be taken up and stored in a specimen and leading to iron storage diseases [43].

A carbon coating as is present in the ICNP used in our study has been shown to decrease toxicity significantly. In a study treating in vitro cultured pig kidney epithelial cells with carbon coated Fe_7_C_3_ ICNP, a high intracellular uptake without any negative impact on cell mitosis, progression on the cell cycle or ability to proliferate was demonstrated [44].

However, a study conducted on human mesenchymal stem cells using ICNPs, still showed a dose-dependent reduced cell viability, ranging from 1 to 50 μg/ml. Also, an increased stiffness of the cells due to the NPs adhering to the cellular membrane could be observed even at the lowest concentration of 1 μg/ml [45]. Whether the same effect could be observed using ICNPs, remains to be tested.

There are several studies assessing the short-term effect of NPs on a cellular level and in complex living organisms such as adult mice. There, no toxic effects could be discerned. A long-term study observing the influence of functionalized, platinum-spiked Fe_3_C@C mice over the course of one year, has also shown no tissue alterations or changes in organ function with the NPs still being present inside the tissue [25]. It needs to be taken into account that the investigated specimen were not developing organisms. Various studies exist, discussing the adverse toxic effects of every-day nanoparticles on embryos, with some NPs containing carbides leading to developmental issues and are linked to certain problems in adulthood [20]. Further studies including a higher number of subjects need to be done to determine the long term effect of ICNPs on developing organisms.

### 4.1 Limitations and next steps

Some limitations need to be highlighted concerning this study. Only a small number of animals have been used with three chicken embryos dedicated to each concentration and nanoparticle amount. Due to rules of animal welfare, the 3R principles (reduce, replace, refine) must be applied and we believe that with the applied study design, we were able to demonstrate the main aims of our study. However, we cannot exclude that using more animals in group and comparing more dosages of NP injections, clearer statements regarding survival rate and dose dependency could be made (SI Fig. 2).

As mentioned, the needles used for injections were self-pulled glass capillaries. Accidental air injection cannot be excluded as a confounding factor regarding death rate, as well as volume overload and consecutive heart failure. Worth considering would be administering the ICNPs via an injection into the albumin instead of direct injection into a blood vessel. A study performed by Kurantowicz et al. investigating the toxicity of six types of nanoparticles on chicken embryos by injecting the experimental solution into the egg albumin proves this administration effective [46].

The exact time of death is also not definitive, as the chicken embryos have not been observed for 22 h of 24 h before fixation. Interesting to see would also be the further development of the chicken embryos having survived 24h after injection and for how long the ICNPs would still be detectable and if the biodistribution changes with time. As an intermediate step, the influence of carbon-coated iron carbide nanoparticles on embryological mesenchymal stem cells and the gene regulation of crucial developmental genes might be of interest.

Due to the artefacts arising from possible susceptibility effects and field inhomogeneities introduced by the nanoparticles, the interpretation of the images is challenging and less reliable, particularly considering the small size of the specimen. A much lower dose of injected ICNPs might lead to better visualization of the different structures. Further studies investigating lower dosages and finding a threshold in small specimen such as chicken embryos could be useful. For better assessment in MRI, ultra short echo time (UTE) sequences could be applied, as a study conducted with IONPs by Boss et al. suggested [17].

The focus of this study was on the qualitative and semi-quantitative An Everhart-Thornley assessment of the biodistribution, further studies using a different scan protocol need to be made to further shed light into the changes in transverse relaxation time, providing a quantitative readout.

## 5 Conclusion

This study provided early insights into the in vivo performance of ICNPs by investigating the biodistribution and potential impacts in developing chicken embryos. The magnetic properties of ICNPs in the accumulated tissues led to strong hypointensities visible on the MRI scans, especially in the abdominal and developing brain area. However, susceptibility artefacts and field inhomogeneities made a precise delineation of the biodistribution in MRI challenging, particularly at higher dosages. Through histological and EDX analysis we could confirm nanoparticle uptake within various tissues, including neural structures, proving their ability to cross the blood-brain barrier during embryological development. The survival rates following injection of ICNPs were significantly lower than expected, with possible confounding causes including volume overload, air embolisms, and nanoparticle aggregation inside blood vessels, which may have led to circulatory collapse. Our findings suggest that ICNPs show promise as a contrast agent and for potential theragnostic purposes, but the substantial mortality rates call for further testing. Next steps include focusing on dose optimization, alternative delivery methods, and the long-term impact of ICNPs when administered during early developmental stages, especially considering their accumulation in sensitive tissues such as the brain and retina. Furthermore, improving imaging techniques by using UTE sequences or higher-field MRI systems, may enable a better visualization of the NPs. Overall, while ICNPs show promise as multifunctional nanomaterials, their effects on developing organisms must be further tested to ensure safety in future biomedical applications.

## Confirmation

All authors have approved the final version of the manuscript.

## Disclosure

All authors confirm that they did not use any AI Tools for manuscript preparation.

## Funding

There was no particular grant for this study. It has been funded by the institutional budget.

## CRediT authorship contribution statement

**Désirée Schibler:** Conceptualization, Data curation, Formal analysis, Investigation, Methodology, Writing – original draft, Writing – review & editing. **Anna Landsmann:** Data curation, Writing – review & editing. **Petra Wolint:** Conceptualization, Data curation, Writing – review & editing. **Oscar Cipolato:** Data curation, Investigation, Methodology, Writing – review & editing. **Fabian Starsich:** Investigation, Methodology, Writing – review & editing. **Pietro Giovanoli:** Supervision, Writing – review & editing. **Inge Herrmann:** Conceptualization, Supervision, Writing – review & editing. **Andreas Boss:** Conceptualization, Methodology, Supervision, Writing – review & editing. **Johanna Buschmann:** Conceptualization, Data curation, Investigation, Methodology, Project administration, Resources, Supervision, Validation, Writing – review & editing.

## Declaration of competing interest

The authors declare that they have no known competing financial interests or personal relationships that could have appeared to influence the work reported in this paper.

## Data Availability

Semi-quantitative data of contingency tables as well as MRI images can be provided upon request.
