# Supplementary material for: Iron nanoparticles – biodistribution in the chicken embryo model
Source: Biochem Biophys Rep. 2026 Mar 30;46:102567. doi: 10.1016/j.bbrep.2026.102567 (PMC13068616; doi:10.1016/j.bbrep.2026.102567)
Supplement: Multimedia component 1 [file mmc1.pdf]

Supporting Information

Iron Nanoparticles – biodistribution in the chicken embryo model

Désirée Schibler[1], Anna Landsmann[2], Petra Wolint[1], Oscar Cipolato[3], Fabian Starsich[3], Pietro Giovanoli[1], Inge Herrmann[3], Andreas Boss[4,]* and Johanna Buschmann[1,]*

[1] Department of Plastic Surgery and Hand Surgery, University Hospital Zurich, Zurich, Switzerland

[2] Kantonsspital Baden, Im Ergel 1, 5404 Baden

[3] Nanoparticle Systems Engineering Laboratory, Department of Mechanical and Process Engineering, ETH Zurich, Zurich, Switzerland

[4] GZO AG Spital Wetzikon, Wetzikon, Switzerland

Corresponding author: Johanna Buschmann (Johanna.Buschmann@usz.ch)

* These authors contributed equally as senior scientists.

SI Table 1 Semi-quantitative scoring of MR signal intensity for all tested specimen

Group	None	Low	Middle	High	Very High	Total
Bubbles	3	0	0	0	0	**3**
Control	3	0	0	0	0	**3**
**300µg**						**9**
H&H24 300 µg	0	2	1	0	0	**3**
CNS		2	1	0	0	**3**
Abdomen		1	2	0	0	**3**
H&H25 300 µg	0	2	1	0	0	**3**
CNS		2	1	0	0	**3**
Abdomen		2	0	1	0	**3**
H&H26 300 µg	0	0	2	1	0	**3**
CNS		0	2	1	0	**3**
Abdomen		0	1	2	0	**3**
**600µg**						**9**
H&H24 600 µg	0	1	0	2	0	**3**
CNS		1	0	2	0	**3**
Abdomen		0	1	1	1	**3**
H&H25 600 µg	0	0	1	2	0	**3**
CNS		0	2	1	0	**3**
Abdomen		0	1	2	0	**3**
H&H26 600 µg	0	0	1	1	1	**3**
CNS		0	1	1	1	**3**
Abdomen		0	0	2	1	**3**
**Total 300 µg**	0	4	4	1	0	**9**
CNS		4	4	1	0	**9**
Abdomen		3	3	3	0	**9**

**Total 600 µg**	0	1	2	5	1	**9**
CNS		1	3	4	1	**9**
Abdomen		0	2	5	2	**9**
**Total Fe_3C**	**0**	**5**	**6**	**6**	**1**	**18**
CNS		**5**	**7**	**5**	**1**	**18**
Abdomen		**3**	**5**	**8**	**2**	**18**

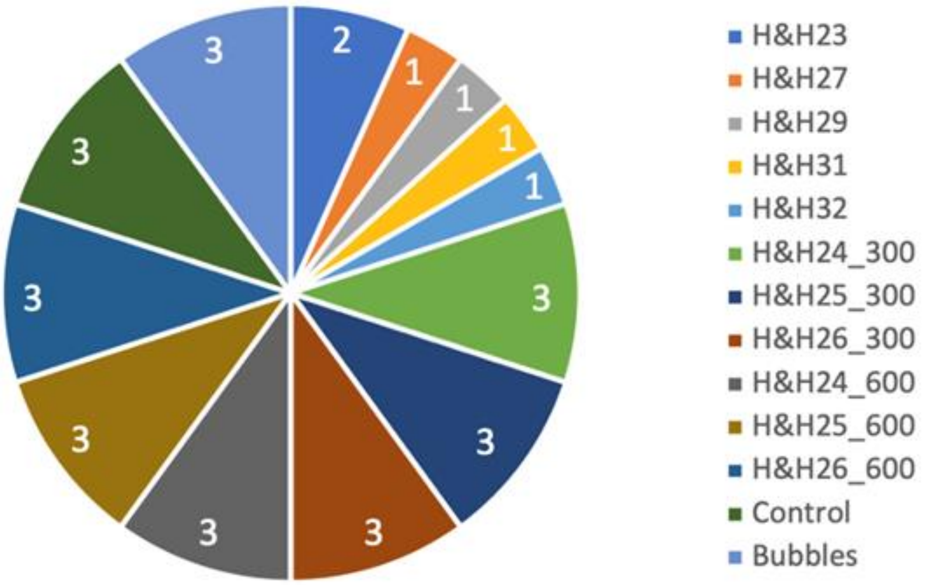

SI Figure 1 Schematic overview of the quantity and ages (H&H stage) of chicken embryos used in this study**.**

A)

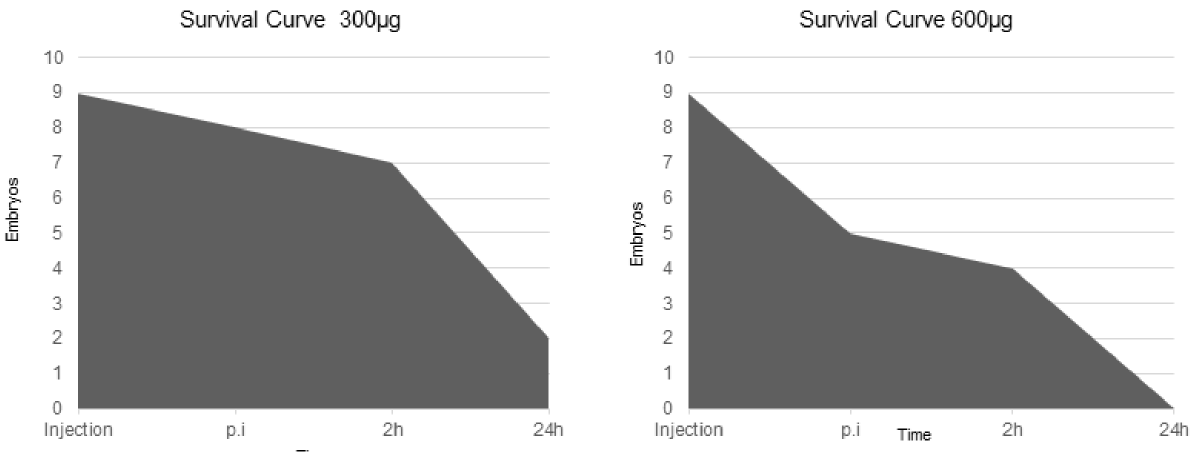

B)

**Mortality**	Post injection	Within 2 h	Within 24 h	After 24 h	Total
300 µg	1	1	5	2	9
600 µg	4	1	4	0	9
Total	5	2	9	2	18

SI Figure 2: Survival curves of the cohort with 300 µg ICNPs injected on the left and 600 µg injected on the right over the course of 24 h. The survival counts for the 300 µg cohort were 9 at injection, 8 post injection, 7 after 2h and 2 after 24 h. The survival counts for the 600 µg cohort are 9 at injection, 5 post injection, 4 after 2h and 0 after 24 h, respectively (**A**). Crosstable for mortality with contingency coefficient 0.422 and p = 0.271. The mortality was not significantly different between the two dosages (**B**).

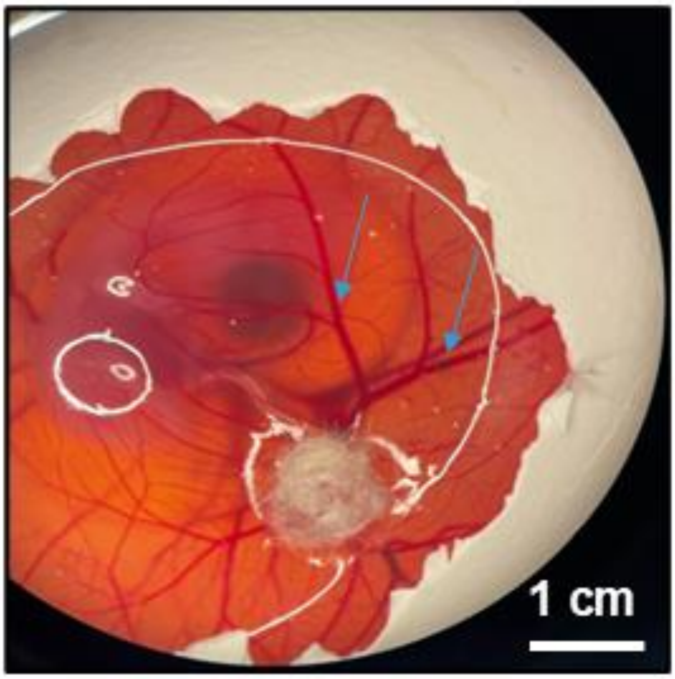

SI Figure 3: Example of visibly clustered ICNPs inside blood vessels in an embryo used for injection training.
